# Alginate-Chitosan Hydrogels Containing shRNA Plasmid for Inhibition of CTNNB1 Expression in Breast Cancer Cells

**DOI:** 10.3390/gels9070541

**Published:** 2023-07-04

**Authors:** Birnur Cömez, Suna Özbaş

**Affiliations:** Department of Pharmaceutical Biotechnology, Faculty of Pharmacy, Marmara University, İstanbul 34854, Turkey

**Keywords:** sodium alginate, chitosan, breast cancer, shRNA, hydrogel

## Abstract

The hydrogels prepared with alginate and chitosan polymers were prepared to deliver the shRNA-encoding plasmid (pshRNA) to MDA-MB-231 cells for the inhibition of β-catenin (CTNNB1), which was reported to be overexpressed in breast cancer. Polyion complex hydrogels prepared using sodium alginate and chitosan were characterized by Fourier transform infrared spectrometry (FTIR) analysis, scanning electron microscope (SEM) analysis, swelling, and degradation properties. After the release properties and serum stability of pshRNA-loaded hydrogels were determined, their cytotoxicity, transfection efficacy, and effects on CTNNB1 expression were investigated in MDA-MB-231 cells. All hydrogels were shown to protect pshRNA from the enzymatic activity of serum and to deliver pshRNA to cells efficiently. As a result of transfection studies, pshRNA-loaded hydrogels reduced CTNNB1 expression by up to 30.25%. Cell viability also decreased by 38% in cells treated with 2.5% (*w*/*v*) alginate-chitosan hydrogel containing pshRNA targeting CTNNB1. Alginate-chitosan hydrogels were shown to be a suitable matrix system for local gene delivery.

## 1. Introduction

Breast cancer is the most common malignancy in women worldwide. Approximately 80% of early-stage and nonmetastatic breast cancer patients are treatable. However, advanced breast cancer with distant organ metastases is currently considered incurable with available treatments. The histological and molecular features of breast cancer greatly influence treatment decisions [1]. Nuclear β-catenin has been observed in 63% of breast cancers. As a result of the categorization of breast cancers according to molecular subtype, it was stated that both cytosolic and nuclear β-catenin were observed more frequently in basal-like invasive breast cancers than in other subtypes. Basal-like breast cancers are typically very aggressive and devoid of any targeted therapy because they do not express the estrogen receptor (ER) or human epidermal growth factor 2 (HER2). In this case, Wnt/β-catenin pathway activity appears to be a good therapeutic target for basal-like breast cancer [2].

β-catenin is bound to the cytoplasmic region of type I cadherins in the cell membrane and is required for the structural organization and function of cadherins by binding to the actin cytoskeleton via α-catenin [3]. β-catenin is also a component of the canonical Wnt signaling pathway. Normally, β-catenin is degraded by phosphorylation of serine and threonine residues by the effects of casein kinase I and glycogen synthase kinase 3β (GSK3β) [4]. Wnt proteins inhibit β-catenin phosphorylation and degradation by binding to low-density lipoprotein receptor-associated protein 5/6 (LRP5/6) and frizzled receptor (FRZ). Uncleaved β-catenin then migrates to the nucleus and interacts with T-cell factor/lymphoid enhancing factor (TCF/LEF) to activate target genes related to cell proliferation and survival [5]. The complex of nuclear β-catenin and TCF/LEF acts as a coactivator to stimulate the transcription of cellular oncogenes, including cyclin D1 and c-myc [6].

The RNAi technology used for gene inhibition studies is based on the use of short double-stranded RNA fragments, approximately 21 nucleotides long, complementary to specific regions of mRNA. RNAi molecules such as siRNA and shRNA combine with the RNA-induced silencing complex (RISC) in the cell, causing the targeted mRNA to be fragmented, thereby inhibiting gene expression [7]. The shRNA is delivered into cells as DNA templates using a plasmid or a viral vector. The transcript, with a short hairpin loop ranging from fifty to one hundred nucleotides in length, is formed in the cell. Unlike siRNAs, shRNAs need to be processed within target cells before binding to RISC. It is processed in the nucleus by Drosha and then transported to the cytoplasm by Exportin 5. In the cytoplasm, the Dicer complex splices shRNA to form double-stranded RNA fragments with a 2-nucleotide overhang, such as siRNA [8].

RNAi inhibition of mutated oncogenic genes, which play a major role in tumorigenesis and tumor progression, has been seen as promising for cancer treatment [9]. Inhibition of β-catenin by RNAi resulted in a significant decrease in estrogen receptor alpha (ERα) mRNA and protein levels in MCF-7, T-47D, and BT-474 breast cancer cells, while also leading to a significant reduction in the growth of MCF-7 cells both in the presence and absence of estradiol [10]. It was observed that tumors formed in mice with the triple-negative breast cancer cell line, HCC38, in which β-catenin expression was inhibited by the shRNA lentiviral vector, were smaller in size, and the tumor growth rate was significantly reduced. However, breast cancer cells with inhibited expression of β-catenin were found to be more sensitive to the chemotherapeutic agents, doxorubicin, and cisplatin [11]. The use of RNAi is limited due to its very short RNA half-life and rapid removal from the body. Although RNAi molecules can be potential therapeutic agents for various diseases, an appropriate carrier system is required for these molecules to be effective [12]. Hydrogels can be used to deliver genetic material locally, provide sustained release, and maintain high concentrations. Hydrogels provide an environment for cell adhesion and migration with their high-water content and similar physical properties to many tissues [13]. The cross-links found in the structure of hydrogels allow the encapsulation of bioactive molecules [14].

Many synthetic and natural polymers have been studied to produce hydrogels, but natural polymers have been shown to have both low toxicity and a better effect on cell division, adhesion, and migration compared to synthetic ones [15]. Chitosan, produced from chitin by deacetylation, is a biocompatible polymer besides being adhesive due to its positive charge at physiological pH [16]. Chitosan is frequently preferred in drug and gene delivery and tissue engineering applications due to its cationic structure, low toxicity, low immunogenicity, biodegradability, mucoadhesive, and antibacterial properties [17,18]. Alginate is a linear copolymer containing blocks consisting of (1,4)-linked βD-mannuronate (M) and α-L-glucuronate (G) residues. Alginates are water-soluble and biocompatible polyanionic polysaccharides produced by brown algae and bacteria [19]. Due to its nontoxicity and biocompatibility, alginate is often used in wound dressing and tissue engineering applications to produce a matrix to which cells can attach and proliferate [20,21]. The alginate matrix was shown to be resistant to cell adhesion and proliferation due to its highly hydrated anionic surface [22]. These properties can be improved by combining them with different biomaterials [21]. It has been suggested that the hydrogel structure obtained by the cross-linking of alginate and chitosan will be more stable, and cell-matrix interaction will be improved [22]. Rassu et al. reported that a hydrogel comprised of alginate and chitosan could be achieved by adjusting the pH of the mixing solution without adding calcium cations [23]. When the pH of the aqueous solution is lower than 6.5, chitosan is soluble and able to form a polyion complex hydrogel with polyanionic alginate thanks to its positively charged amino groups by protonation [24,25]. The mixing of polycation and polyanion solutions often leads to inhomogeneous precipitation. The cross-link density is low due to the limited number of charged ion pairs involved in the reaction. By adding chitosan as a powder to the alginate solution, it is ensured that the chitosan is dispersed homogeneously, and the cross-link density is increased by increasing the interaction of the oppositely charged ions of the chitosan and alginate. Complexation of the two polyelectrolytes is induced by decreasing the pH [25].

In this study, we intended to evaluate the effect of CTNNB1 gene silencing with pshRNA-loaded alginate-chitosan hydrogels. We prepared the hydrogels using different concentrations of sodium alginate and chitosan. After characterization of the hydrogels, β-catenin expression levels and cell viability were evaluated in triple-negative breast cancer cells, MDA-MB-231. The results of this study are expected to contribute to the design and development of biocompatible alginate-chitosan-based matrix delivery systems for gene therapy applications.

## 2. Results and Discussion

### 2.1. Preparation and Control of pshRNA-Loaded Alginate-Chitosan Hydrogels

Alginate-chitosan hydrogels were prepared only with pH change without the use of a cross-linking agent in a 1:1 polymer ratio containing sodium alginate and chitosan at concentrations of 2%, 2.5%, and 3%. The same amount of 8% glacial acetic acid and 1M NaOH solution was used in the hydrogels, and pH of the hydrogels was adjusted to 5–7 (Table 1).

**Figure 1:**
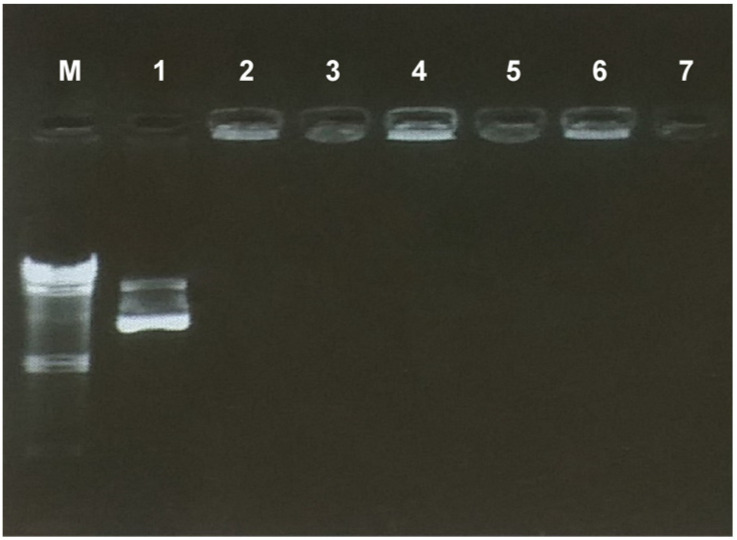
Agarose gel image of the hydrogels with different polymer concentrations. M-Lambda DNA/Hind III marker, 1. Free pshRNA-CTNNB1, 2. Nac1 (2%) with pshRNA, 3. Nac1 (2%), 4. Nac2 (2.5%) with pshRNA, 5. Nac2 (2.5%), 6. Nac3 (3%) with pshRNA, 7. Nac3 (3%).

Agarose gel electrophoresis was used to control whether the hydrogels contained pshRNAs. Figure 1 shows that pshRNA was entrapped in the alginate-chitosan hydrogels. pshRNA-loaded hydrogels appear brighter than blank hydrogels in wells because pshRNAs included in hydrogels were detained within wells. The positively charged amino groups in the chitosan structure can interact with nucleic acids through electrostatic forces [18]. The electrostatic interaction between the DNA and chitosan within the hydrogel network induced condensation of the pshRNA.

### 2.2. Characterization of the Hydrogels

#### 2.2.1. FTIR Analysis

**Figure 2:**
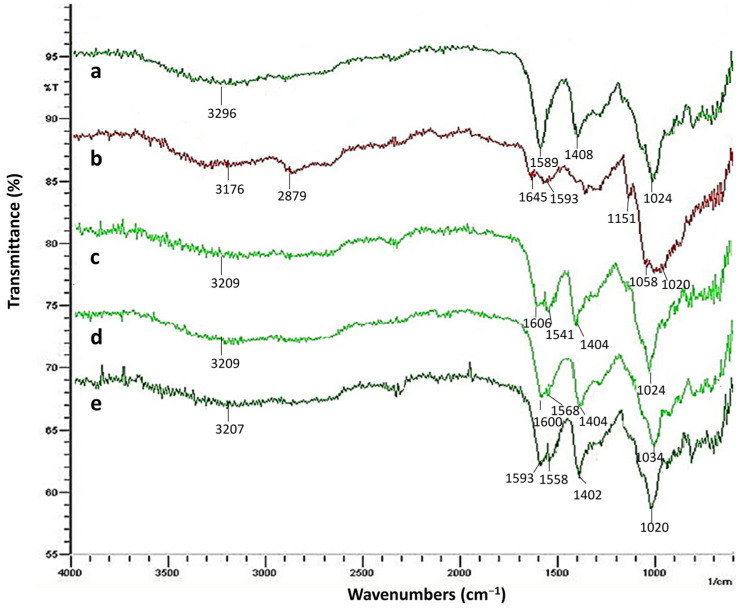
FTIR spectra of chitosan, sodium alginate, and pshRNA-loaded alginate-chitosan hydrogels. (a) Sodium alginate; (b) chitosan; (c) pshRNA + Nac1; (d) pshRNA + Nac2; (e) pshRNA + Nac3.

FTIR spectroscopy was used to investigate the interactions between the polymers in the hydrogels. FTIR results for chitosan, sodium alginate, and alginate-chitosan hydrogels are shown in Figure 2.

The spectra of sodium alginate, chitosan, Nac1, Nac2, and Nac3 hydrogels indicated the broadband in the region 3296–3176 cm^−1^ corresponding to O-H stretching. The characteristic peaks of chitosan were observed at 2879 cm^−1^ (C-H stretching), 1645 cm^−1^ (C=O stretching of amide I), 1593 cm^−1^ (N-H bending peak of amide II), and 1058–1020 cm^−1^ (C-O stretching). The characteristic peaks of the sodium alginate appeared at 1589 cm^−1^ (-COO asymmetric stretching) and 1408 cm^−1^ (-COO symmetric stretching peak). The asymmetric band of carboxylate anions at 1589 cm^−1^ shifted to higher wavelengths in the spectra of the alginate-chitosan hydrogels [16]. Amide-I peak disappeared and amid-II peak became more apparent in the spectra of Nac1, Nac2, and Nac3 hydrogels [25]. These changes suggested an interaction between the negatively charged carboxyl groups of sodium alginate and the positively charged amino groups of chitosan.

#### 2.2.2. SEM Analysis

**Figure 3:**
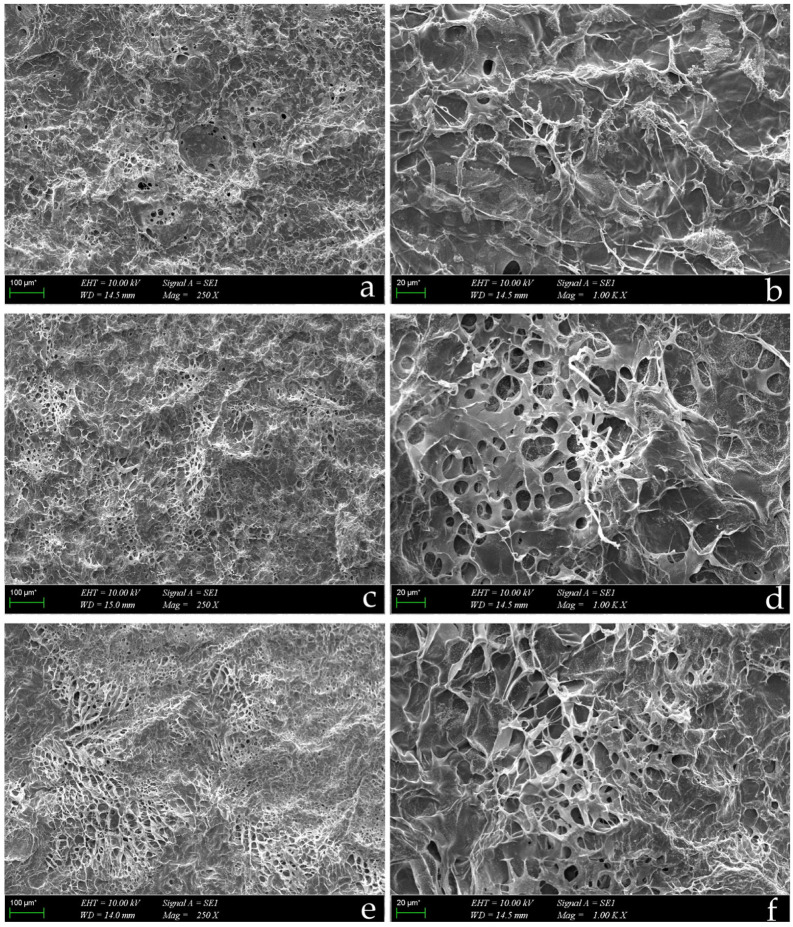
SEM images of the alginate-chitosan hydrogels at different magnifications. (**a**) Nac1 (250×); (**b**) Nac1 (1000×); (**c**) Nac2 (250×); (**d**) Nac2 (1000×); (**e**) Nac3 (250×); (**f**) Nac3 (1000×).

The surface morphologies of the hydrogels were observed by SEM. SEM images of the plasmid-loaded hydrogels are shown in Figure 3. All hydrogels had an interconnected, porous 3D network. The lyophilized hydrogels were highly porous, allowing the passage of pshRNA to the application area. It was seen that the porosity of hydrogels increased as the polymer concentration increased. The higher porosity causes an increase in the rate of drug release [26].

#### 2.2.3. Swelling Properties

**Figure 4:**
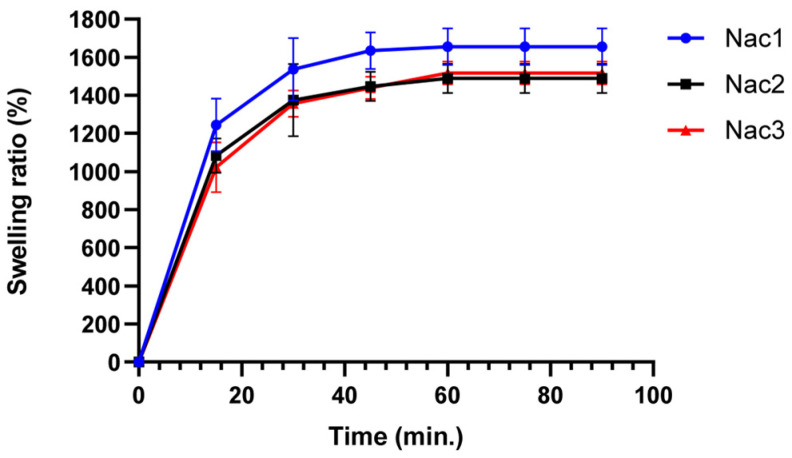
The time-dependent swelling capacity of pshRNA-loaded alginate-chitosan hydrogels.

Hydrogels are materials that can hold water and swell due to the cross-linking network in their structures [27]. The hydrogels containing pshRNA were able to absorb water approximately 1400–1600% of their weight (Figure 4). The hydrogel with the highest water absorption capacity is the Nac1 hydrogel, which contains 2% (*w*/*v*) chitosan-alginate. The swelling profiles of the Nac2 and Nac3 hydrogels were found to be very close to each other. Ionically cross-linking hydrogels are strongly affected by pH changes [28]. The pH value of the hydrogels increased as the polymer concentration increased. Nac1 hydrogel had both the lowest polymer ratio and the lowest pH value. pH is a factor affecting the swelling properties of chitosan. At low pH, the maximum swelling rate of chitosan increases due to the protonated free amino groups [29]. The increase in charged ionic groups in hydrogels will increase the osmotic pressure and charge repulsion, thus increasing the degree of swelling [30].

#### 2.2.4. Degradation Properties

**Figure 5:**
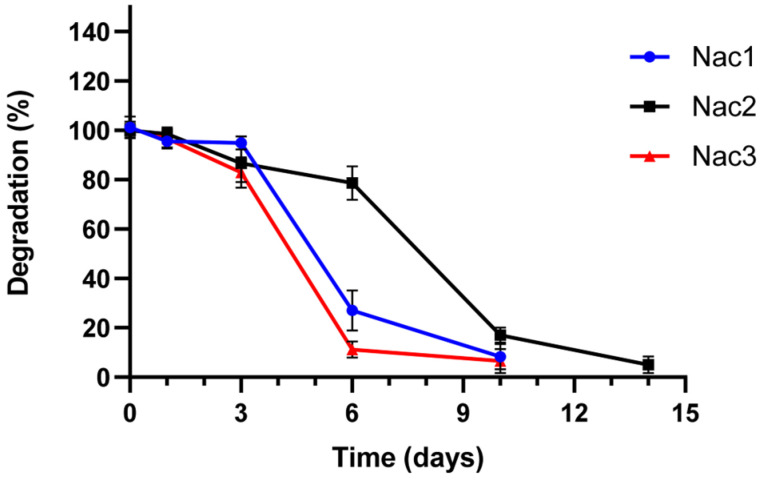
Degradation of the hydrogels in PBS (pH 7.4) as remaining weight (%).

The weight loss (%) of Nac1, Nac2, and Nac3 hydrogels was calculated to determine the degradation properties of hydrogels in PBS (pH 7.4) at 37 °C. Hydrogels started to degrade after day 1 (Figure 5). Nac1 and Nac3 hydrogels were completely degraded within 10 days. On the other hand, Nac2 hydrogel lost more than 80% of its weight on Day 10. Among the hydrogels, Nac2 hydrogel endured degradation conditions for the longest time. The degradation rates of mechanically more durable hydrogels are also slower [31]. The mechanical strengths of Nac1 and Nac3 hydrogels were lower compared to the Nac2 hydrogel.

#### 2.2.5. Serum Stability

**Figure 6:**
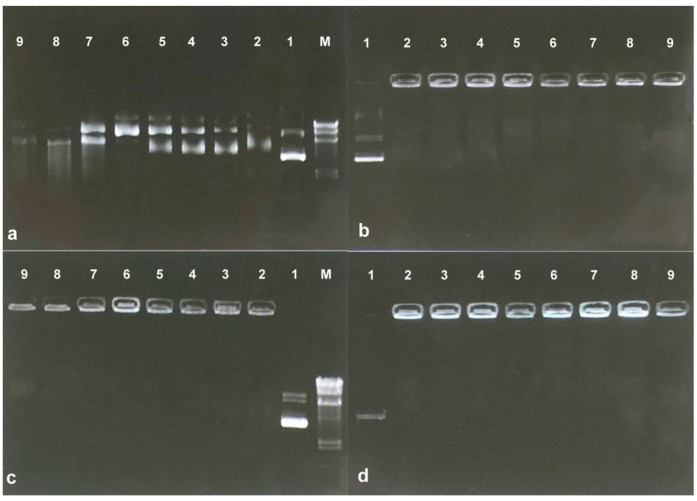
Agarose gel image of the serum stability study. (**a**) pshRNA-CTNNB1; (**b**) Nac1; (**c**) Nac2; (**d**) Nac3. M-Lambda DNA/Hind III marker, 1–Free pshRNA, 2–0 min, 3–15 min, 4–30 min, 5–1 h, 6–4 h, 7–24 h, 8–48 h, 9–72 h.

The presence of serum nucleases in the extracellular environment causes rapid degradation of the genetic material [32]. The stability of pshRNA-loaded hydrogels incubated in PBS (pH 7.4) with 10% FBS was examined by agarose gel electrophoresis. The results regarding the stability of free pshRNA and pshRNA-loaded hydrogels are shown in Figure 6. Free pshRNA started to disintegrate at 0 min. However, all hydrogel formulations protected pshRNA from the enzymatic degradation of serum for 72 h. It has been shown previously that DNA condensation by the positively charged polymer delays DNA mobility in electrophoresis and inhibits enzyme cleavage [33].

#### 2.2.6. Release Profiles

**Figure 7:**
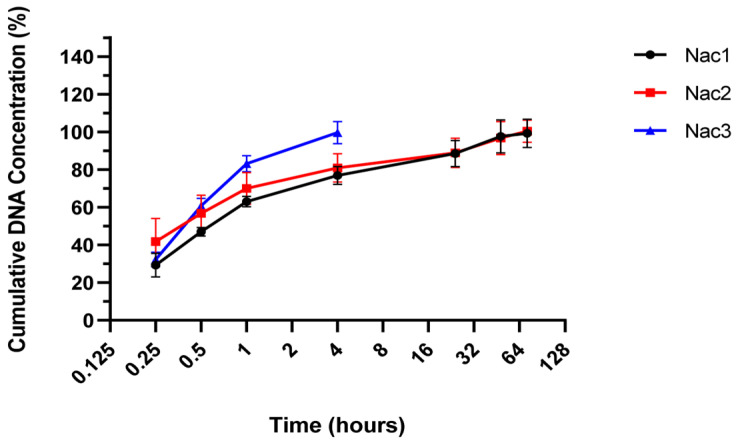
The release profiles of alginate-chitosan hydrogels in PBS (pH 7.4) at 37 °C.

The release of pshRNA from Nac3 hydrogel was completed within 4 h, while those from Nac1 and Nac2 hydrogels were completed within 72 h (Figure 7). The release properties of Nac1 and Nac2 hydrogels appeared to be similar. Alginate-chitosan hydrogels were observed to have a burst effect on the release profile. As a result of the higher pH value of the Nac3 hydrogel than the others, the cross-link density in the structure of the hydrogel will be less because the increase in the pH value causes a decrease in the charged amino groups [24]. Plasmid release from the hydrogel varied depending on the physical structure and degradation of the hydrogel. Plasmid release was slower in mechanically stable hydrogels, as seen in previous studies [14,34,35].

### 2.3. Cellular Uptake and CTNNB1 Protein Levels

**Figure 8:**
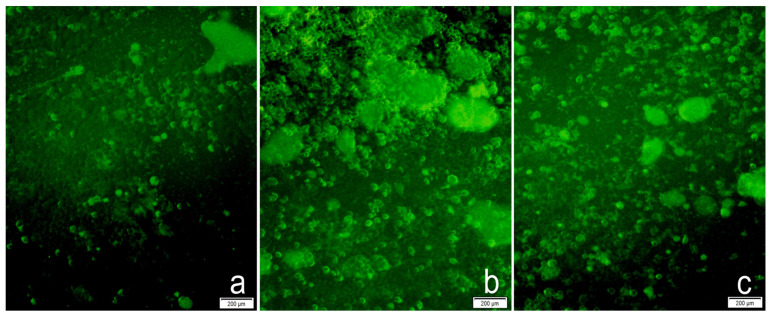
Detection of GFP expression in MDA-MB-231 cells 72 h after transfection with pshRNA-loaded alginate-chitosan hydrogels by fluorescence microscope (10× magnification). (**a**) Nac1 hydrogel with pshRNA-CTNNB1; (**b**) Nac2 hydrogel with pshRNA-CTNNB1; (**c**) Nac3 hydrogel with pshRNA-CTNNB1.

Cellular uptake of pshRNA was observed by a fluorescence microscope thanks to GFP expression by cells. As shown in Figure 8, MDA-MB-231 cells were transfected successfully with alginate-chitosan hydrogels. The GFP expression was observed to be more intense in Nac2 and Nac3 hydrogels than in Nac1. This may be related to the increased amount of chitosan and its ability to bind positively charged amino groups to negatively charged nucleic acids via electrostatic interaction. Primary amines in the chitosan structure are protonated at a slightly acidic pH, thus causing the positively charged amino groups to interact with nucleic acids via electrostatic forces. Additionally, because the cell membrane and nuclear membrane are negatively charged, their interaction with positively charged chitosan contributes to the cellular uptake of nucleic acids [18]. Chitosan is adhesive to cells due to its polycationic nature. The chitosan-alginate hybrid material has been shown to inherit cellular adhesive properties [36]. Due to the adhesive nature of chitosan, chitosan-based particulate systems carrying genetic material enable enhanced transfection efficiency to recipient cells [37].

**Figure 9:**
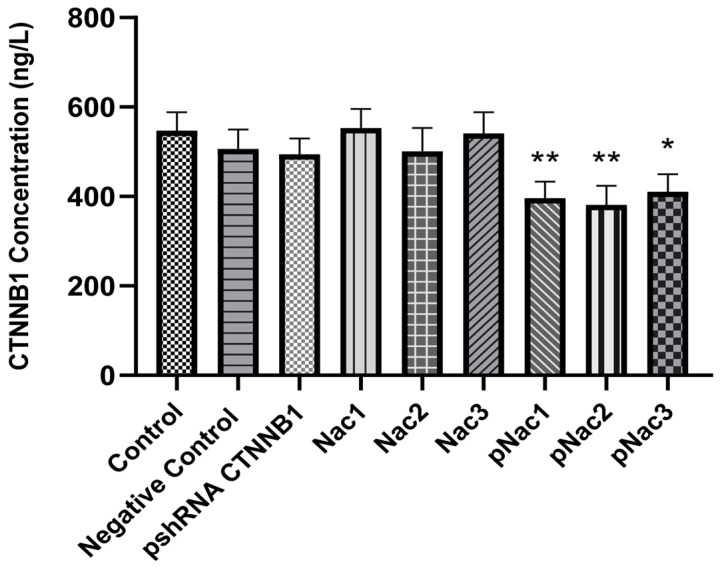
Effect of alginate-chitosan hydrogels on CTNNB1 expression in MDA-MB-231 cells. Results were shown as mean ± SD (n = 3) and 1µg of free pshRNA-CTNNB1 was given to the cells (negative control: Nac2 hydrogel with pshRNA-NC, Nac1: 2% alginate-chitosan hydrogel, Nac2: 2.5% alginate-chitosan hydrogel, Nac3: 3% alginate-chitosan hydrogel, pNac1: Nac1 hydrogel with pshRNA-CTNNB1, pNac2: Nac2 hydrogel with pshRNA-CTNNB1, pNac3: Nac3 hydrogel with pshRNA-CTNNB1). * indicates *p* < 0.05 and ** indicates *p* < 0.01 according to the control group.

After transfection of pshRNA-loaded hydrogels, CTNNB1 protein levels in cells were determined by an ELISA assay. CTNNB1 protein levels in MDA-MB-231 cells 72 h after transfection are shown in Figure 9. Nac2 hydrogel containing pshRNA provided the highest suppression of CTNNB1 expression at 30.25%. Then, Nac1 hydrogels at 27.3% and Nac3 at 25% suppressed CTNNB1 expression. Blank hydrogels had no significant effect on gene expression. All hydrogels containing pshRNA-CTNNB1 significantly decreased CTNNB1 expression compared to the control group (*p* < 0.05). Although the highest suppression was obtained with the Nac2 hydrogel, similar results were obtained with all hydrogels.

### 2.4. Cell Proliferation

**Figure 10:**
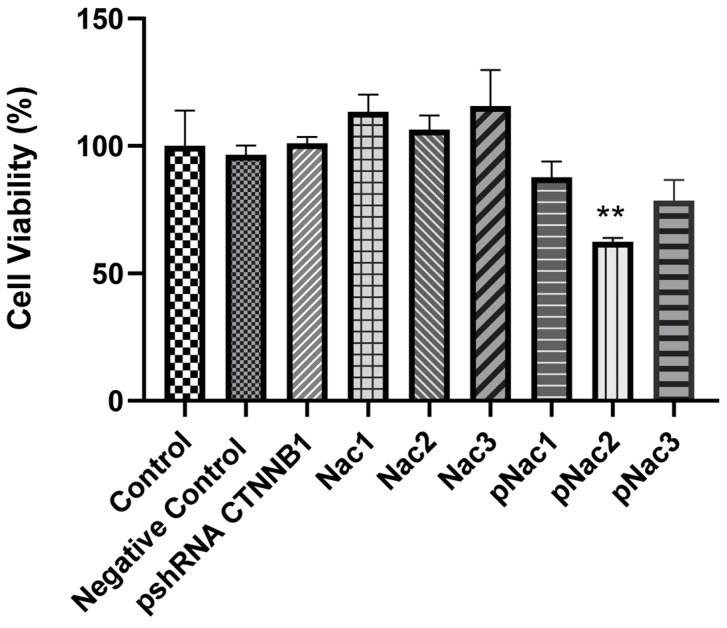
Cell viability (%) results in MDA-MB-231 cells 72 h after treatment with alginate-chitosan hydrogels. Results were shown as mean ± SD (n = 3) and 1µg of free pshRNA-CTNNB1 was added to the cells (negative control: Nac2 hydrogel with pshRNA-NC, Nac1: 2% alginate-chitosan hydrogel, Nac2: 2.5% alginate-chitosan hydrogel, Nac3: 3% alginate-chitosan hydrogel, pNac1: Nac1 hydrogel with pshRNA-CTNNB1, pNac2: Nac2 hydrogel with pshRNA-CTNNB1, pNac3: Nac3 hydrogel with pshRNA-CTNNB1). ** indicates *p* < 0.01 according to the control group.

The cytotoxic effect of blank hydrogels and hydrogels containing pshRNA in MDA-MB-231 cells was determined by MTT assay. The results obtained 72 h after the hydrogels were applied to the cells are shown in Figure 10. The viability rates of cells treated with pshRNA-loaded Nac1, Nac2, and Nac3 hydrogels were 87.6% ± 7.7%, 62.4% ± 1.9%, and 78.5% ± 10%, respectively. Blank hydrogels and pshRNA NC-loaded hydrogels had no reducing effect on cell viability. Only the pshRNA-loaded Nac2 hydrogel significantly reduced cell viability compared to the control group (*p* < 0.01). Other pshRNA-loaded hydrogels also seem to decrease cell viability compared to the control group, but the difference between them is not statistically significant. At the same time, Nac2 was the hydrogel with the highest decrease in CTNNB1 levels. Zhang et al. strongly suppressed β-catenin expression using siRNA in MA-891 cells obtained from TA2 mice with spontaneous breast cancer and thus showed that cell cycle and proliferation were inhibited [38]. Ashaie et al. showed that the delivery of CTNNB1 siRNA with carbonate apatite nanoparticles significantly reduced cell viability in 4T1 breast cancer cells [39]. Our results are compatible with the earlier papers mentioned above.

## 3. Conclusions

Our study has shown that alginate-chitosan hydrogels can be used to deliver shRNA plasmids to cells for the inhibition of overexpressed genes leading to cancer. The degradation and release properties of the alginate-chitosan hydrogels could be controlled by changing the concentrations of the polymers and pH, and thus the efficiency could also be changed. The rate of release increased due to the loss of hydrogel integrity. Nac2 hydrogel prepared with 2.5% sodium alginate and chitosan was determined to be the most appropriate formulation regarding physicochemical properties and transfection efficiency. This study showed that the prepared alginate-chitosan hydrogels could be suitable biomaterials for local gene delivery.

## 4. Materials and Methods

### 4.1. Materials

Chitosan (low molecular weight, 75–85% degree of deacetylation) was purchased from Sigma Aldrich (St. Louis, MO, USA). Sodium alginate (low viscosity, 1.56 ± 0.01 cP, 2% in distilled H_2_O (25 °C)) was provided by İlko Pharmaceuticals (İstanbul, Turkey). We used the Human CTNNB1 ELISA Kit (BT-Lab, Shanghai, China), MTT (3-(4,5-dimethylthiazol-2-yl)-2,5-diphenyltetrazolium bromide) Cell Proliferation Kit (Roche, Mannheim, Germany), PureLink HiPure Plasmid Maxiprep Kit (Invitrogen, Waltham, MA, USA). Dulbecco’s Modified Eagle Medium (DMEM) and fetal bovine serum (FBS) were obtained from PAN Biotech (Aidenbach, Bavaria, Germany). All other chemicals used were molecular-grade.

### 4.2. Transformation and Isolation of shRNA Plasmid

pshRNA-CTNNB1 (GenePharma, Shanghai, China) transcribes stem-loop structured shRNA targeting CTNNB1 mRNA under the control of the SV40 promoter in eukaryotic cells. The type of shRNA expression vector is pGPU6/GFP/Neo. The plasmid structure, which is 5117 bp in size, contains the Kanamycin/Neomycin antibiotic resistance gene and the GFP reporter gene. The target sequence of pshRNA-CTNNB1 is GCTTGGAATGAGACTGCTGAT. The target sequence was selected against human CTNNB1 mRNA (Accession number NM_001098210.2). pshRNA-NC (target sequence: TTCTCCGAACGTGTCACGT) as a negative control was also synthesized and purified by Genepharma (Shanghai, China).

pshRNA-CTNNB1 and pshRNA-NC were transformed into E. coli DH5-alpha bacterial strains. Transformed cells were grown in LB broth containing Kanamycin (100 µg/mL). The plasmid was isolated according to protocol using the PureLink HiPure Plasmid Maxiprep Kit (Invitrogen, USA). DNA concentration was measured by UV-vis spectrophotometer (Shimadzu Biospec 1601, Shimadzu Scientific Instruments, Kyoto, Japan) at 260 nm. The purity of DNA was controlled by agarose gel electrophoresis after ethidium bromide staining.

### 4.3. Preparation of pshRNA-Loaded Alginate-Chitosan Hydrogels

Alginate-chitosan hydrogels were prepared at the concentrations shown in Table 1 using sodium alginate and chitosan polymers. Sodium alginate was dissolved in double-distilled water. Chitosan in powder form was added to the sodium alginate solution and dispersed. The plasmid was added to the chitosan-alginate mixture at a concentration of 50 µg pshRNA/1 g hydrogel. A polyionic gel form was obtained by adding 10 mL of 8% glacial acetic solution to the resulting mixture. Afterward, the pH values of hydrogels were made closer to neutral with 10 mL of 1 M NaOH solution. To check whether the hydrogels contained pshRNA, samples were loaded into agarose gel at 0.8% (*w*/*v*) concentration, and the gels were imaged on a UV transilluminator (Vilber Lourmat, Marne La Vallée, France) after the electrophoresis procedure.

### 4.4. Characterization of pshRNA-Loaded Alginate-Chitosan Hydrogels

#### 4.4.1. FTIR Analysis

The chemical structure of hydrogels was determined by FTIR analysis. The measurements were performed using a Shimadzu FTIR-8400S spectrophotometer (Shimadzu Scientific Instruments, Kyoto, Japan) at a wavelength range of 4000–750 cm^−1^.

#### 4.4.2. SEM Analysis

The morphology of the alginate-chitosan hydrogels was observed by SEM (Zeiss EVO MA10, Oberkochen, Germany). Lyophilized hydrogels were coated with a gold-palladium layer and scanned at 10 kV.

#### 4.4.3. Swelling Study

The swelling capacity of the freeze-dried hydrogels was determined by direct immersion of the hydrogel in phosphate buffer saline (PBS, pH 7.4). The samples were taken out at predetermined time intervals and weighed after excess water was removed using tissue paper. The experiment was performed in triplicate and at room temperature. The swelling ratio of the hydrogels was calculated according to the following equation [20].

(1) $$\mathrm{Swelling}\;\mathrm{ratio}\;(\%)=((W_{t}-W_{d})/W_{d})\;\times \;100\;$$

where W_t_ is the weight of the swollen samples at time t and W_d_ is the weight of the dry samples.

#### 4.4.4. In Vitro Degradation Assay

After the hydrogel samples were weighed into tared containers, they were incubated in PBS at 37 °C in a shaker at 75 rpm. The samples were taken out at certain time intervals, dried, and their weight measured. The degradation was calculated using the following equation [40].

(2) $$\mathrm{Degradation}\;(\%)=(W_{0}-W_{d})/W_{0}\;\times \;100\;$$

W_0_ and W_d_ are the weights before and after incubation, respectively.

#### 4.4.5. Serum Stability Assay

The serum stability assay was performed according to the experiment reported by Salva and Akbuğa [41]. The hydrogels containing 10 µg of pshRNA were suspended in 1 mL of PBS (pH 7.4) with 10% FBS and incubated at 37 °C and 100 μL samples were taken at certain time intervals (0, 15, 30 min; 1, 4, 24, 48, and 72 h), and the reaction was inhibited with 0.5M EDTA. The integrity of pshRNA was controlled by agarose gel retardation assay. The serum stability of free pshRNA was also studied, as described above.

#### 4.4.6. Release Study

Hydrogels containing 20 µg of pshRNA were weighed into sealed sample tubes and incubated in PBS (pH 7.4) at 37 °C with shaking at 75 rpm. At predetermined intervals, the samples were centrifuged at 14,000 rpm for 30 min. The supernatants were collected and replaced with the same volume of fresh buffer [42]. The amount of pshRNA released in the supernatant was measured spectrophotometrically at 260 nm. The supernatant of hydrogel without pshRNA was used as a blank control.

### 4.5. In Vitro Transfection

MDA-MB-231 cells (ATCC HTB-26, Manassas, VA, USA) were cultured in DMEM with 10% FBS, 100 mM L-glutamine, and 100 mM antibiotic solution (penicillin/streptomycin) in an incubator (Sanyo, Osaka, Japan) with 5% CO_2_ at 37 °C. The cells were seeded in a 24-well plate at a cell density of 2.5 × 10^5^ cells/well and incubated overnight. When the cells reached approximately 70% confluence, the medium on the cells was removed, and hydrogels with 1µg pshRNA and without pshRNA were administered to cells with serum-free medium. Nine groups were constituted: control group (untransfected cells), free pshRNA CTNNB1 group, blank Nac1, Nac2, and Nac3 hydrogel groups, and pshRNA CTNNB1-loaded Nac1, Nac2, and Nac3 hydrogel groups, pshRNA NC-loaded hydrogel group. After the cells were incubated for 4–6 h, a fresh medium with 10% serum was added to the cells. After 72 h from transfection of the hydrogels containing pshRNA, the cells were observed using a fluorescence microscope (Olympus CKX41, Tokyo, Japan) to image GFP expression.

### 4.6. Determination of CTNNB1 Expression

CTNNB1 levels in cells after transfection were determined using the ELISA method. The ELISA assay was performed according to the manufacturer’s protocol. CTNNB1 levels were determined using absorbance values spectrophotometrically measured at 450 nm. The decrease in the concentration of CTNNB1 in transfected cells was determined relative to the control group (untreated cells). Three samples were studied for each group.

### 4.7. Cell Proliferation

The effect of prepared alginate-chitosan hydrogels on cell proliferation was determined by MTT assay. MDA-MB-231 cells seeded into 96-well cell culture plates at a concentration of 4 × 10^3^ were incubated overnight at 37 °C and 5% CO_2_. After removing the medium from the cells, the prepared hydrogels were applied to the cells with a serum-free medium. After 4–6 h of incubation, fresh medium with 10% serum was added to each well. After 72 h, 10 µL of MTT reagent was added to the wells, and the cells were incubated for 4–6 h at 37 °C and 5% CO_2_. Formazan crystals formed by living cells in MTT-treated cells were dissolved in the solubilization buffer. Absorbances of the samples were measured spectrophotometrically at 550 nm and 690 nm. Cell viability (%) of the hydrogel-treated groups was determined compared to the control group.

### 4.8. Statistical Analysis

Results were expressed as the mean ± standard deviation (SD). The treatment groups were analyzed using one-way ANOVA with Tukey’s multiple comparisons tests. All analyses were performed using GraphPad Prism 8.0.1 (GraphPad Software, San Diego, CA, USA). *p* < 0.05 was statistically significant.

## Figures and Tables

**Table 1:** Polymer content (*w*/*v*) and pH values of alginate-chitosan hydrogels.

Formulation	Sodium Alginate (%)	Chitosan (%)	pH
Nac1	2%	2%	5.59 ± 0.07
Nac2	2.5%	2.5%	5.98 ± 0.05
Nac3	3%	3%	6.21 ± 0.04

## Data Availability

The datasets generated during and/or analyzed during the current study are available from the corresponding author upon reasonable request.
